# Implications for Training on Smartphone Medication Reminder App Use by Adults With Chronic Conditions: Pilot Study Applying the Technology Acceptance Model

**DOI:** 10.2196/formative.8027

**Published:** 2017-11-10

**Authors:** Daniel Y Park, Elizabeth M Goering, Katharine J Head, Rebecca J Bartlett Ellis

**Affiliations:** ^1^ Department of Communication Studies Indiana University-Purdue University Indianapolis Indianapolis, IN United States; ^2^ Indiana University School of Nursing Indiana University-Purdue University Indianapolis Indianapolis, IN United States

**Keywords:** chronic disease, education of patients, medication adherence, mHealth, mobile apps, smartphone

## Abstract

**Background:**

The majority of middle-aged to older patients with chronic conditions report forgetting to take medications as prescribed. The promotion of patients’ smartphone medication reminder app (SMRA) use shows promise as a feasible and cost-effective way to support their medication adherence. Providing training on SMRA use, guided by the technology acceptance model (TAM), could be a promising intervention to promote patients’ app use.

**Objective:**

The aim of this pilot study was to (1) assess the feasibility of an SMRA training session designed to increase patients’ intention to use the app through targeting perceived usefulness of app, perceived ease of app use, and positive subjective norm regarding app use and (2) understand the ways to improve the design and implementation of the training session in a hospital setting.

**Methods:**

A two-group design was employed. A total of 11 patients older than 40 years (median=58, SD=9.55) and taking 3 or more prescribed medications took part in the study on one of two different dates as participants in either the training group (n=5) or nontraining group (n=6). The training group received an approximately 2-hour intervention training session designed to target TAM variables regarding one popular SMRA, the Medisafe app. The nontraining group received an approximately 2-hour control training session where the participants individually explored Medisafe app features. Each training session was concluded with a one-time survey and a one-time focus group.

**Results:**

Mann-Whitney U tests revealed that the level of perceived ease of use (*P*=.13) and the level of intention to use an SMRA (*P*=.33) were higher in the training group (median=7.00, median=6.67, respectively) than in the nontraining group (median=6.25, median=5.83). However, the level of perceived usefulness (U=4.50, Z=−1.99, *P*=.05) and the level of positive subjective norm (*P*=.25) were lower in the training group (median=6.50, median=4.29) than in the nontraining group (median=6.92, median=4.50). Focus groups revealed the following participants’ perceptions of SMRA use in the real-world setting that the intervention training session would need to emphasize in targeting perceived usefulness and positive subjective norm: (1) the participants would find an SMRA to be useful if they thought the app could help address specific struggles in medication adherence in their lives and (2) the participants think that their family members (or health care providers) might view positively the participants’ SMRA use in primary care settings (or during routine medical checkups).

**Conclusions:**

Intervention training session, guided by TAM, appeared feasible in targeting patients’ perceived ease of use and, thereby, increasing intention to use an SMRA. Emphasizing the real-world utility of SMRA, the training session could better target patients’ perceived usefulness and positive subjective norm that are also important in increasing their intention to use the app.

## Introduction

### Background

Approximately 87.5 million middle-aged to older adults in the United States report having one or more chronic conditions [1], and 68% report not taking or filling medications as prescribed [2]. Medication adherence is critical to reducing negative health-related outcomes such as increased hospitalization, morbidity, and mortality [3-5].

Poor medication adherence among middle-aged to older patients with chronic conditions often stems from forgetting [2]. Complex medication schedules for chronic condition management (ie, polypharmacy) [6] might lead these patients to struggle with remembering medication schedules and, thereby, lead them to poorly adhere to medications [7,8].

Smartphone medication reminder apps (SMRAs) that enable users to (1) record prescribed medication information (eg, medication type and dosing schedule) in the app, (2) receive reminders (eg, alarm and message) from the app at the time to take medications, and (3) monitor medication adherence levels via the app [9], show promise as a way to enhance adherence for middle-aged to older patients with chronic conditions. In an experimental setting, a randomized control trial revealed that the patients with a 3-month SMRA use reported higher levels of medication adherence compared with those without app use [9]. In the real-world setting, an SMRA is available to smartphone owners at little to no cost [10], and there is a high rate of smartphone ownership within the middle-aged to older population. For example, 74% of US adults aged between 50 and 64 years report having smartphones [11], which indicates that an SMRA could be utilized with little to no cost by the majority of these adults. In this regard, the promotion of SMRA use among middle-aged to older patients with chronic conditions could be a feasible and cost-effective way to support their medication adherence.

### Intervention and the Aims of Pilot Study

Patients’ electronic health (eHealth) technology use is likely to be challenged by age-related declines in eHealth literacy [12-14] or ability to incorporate eHealth technology use into health care [15]. As an intervention strategy to promote middle-aged to older adults’ eHealth technology use, existing studies have helped these adults to be capable of using the technologies through training sessions [16,17].

In the same vein, providing training on SMRA use shows promise as an intervention strategy to promote middle-aged to older patients’ app use. Existing studies have indicated the utility of training sessions (eg, demonstrating SMRA features to patients, having patients complete app-related tasks, and providing patients with app-related education materials) in enabling patients to use an SMRA [9,18,19]. However, little attention has been paid to how to design an SMRA training session to be more effective in promoting the patients’ app use, such as which theoretical determinants of app use the training session should focus on to ensure the patients will adopt the use of the app.

The technology acceptance model (TAM) [20,21] provides a useful theoretical framework for informing the design of an SMRA training session, given its focus on the determinants of technology use [20]. Specifically, TAM describes that users’ positive perceptions of technology, such as perceived usefulness and perceived ease of use, might lead to intention to use the technology that might, in turn, lead to actual technology use [22]. Furthermore, TAM describes that technology training might lead users to adopt the use of technology when the training first increases users’ levels of positive perceptions of technology [22]. Although the more recent unified theory of acceptance and use of technology (UTAUT) [23,24] that TAM is incorporated into also describes technology training (ie, facilitating condition) as the determinant of technology use, the UTAUT describes that the training, independently of users’ positive perceptions of technology, might lead users to adopt the use of technology [23,24]. Considering this, when compared with UTAUT, TAM may provide a clearer framework for designing an SMRA training session that aims to ensure the patients’ app use by serving as a blueprint for tracking patients’ progress from receiving an SMRA training session to increasing the levels of positive perceptions of app to adopting the use of the app.

Among the positive perceptions of technology within TAM, perceived usefulness is defined as the degree to which people think their performance could be improved using a technology [20]. In the case of medication adherence, performance in taking medications as prescribed would be improved using the SMRA. Furthermore, existing studies have shown that perceived usefulness is positively related to intention to use a technology [25-27]. On the basis of this prior research, it is likely that training sessions designed to elicit perceived usefulness would also affect intention to use an SMRA.

Another TAM variable related to positive perceptions, perceived ease of use, is defined as the degree to which people think they can use a technology with little effort [20]. Existing studies have shown that perceived ease of use is positively related to intention to use a technology [28-30], and the research shows that an SMRA training session that affects patients’ perceptions of ease at using the app is likely to lead them to use the app.

**Figure 1:**
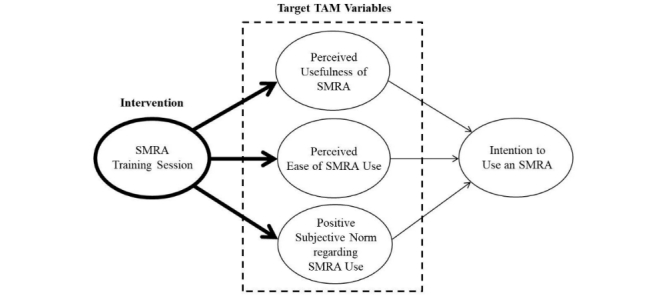
Technology acceptance model (TAM) framework for a smartphone medication reminder app (SMRA) training session.

Existing studies [22,25,31,32] have extended TAM by adding subjective norms or people’s perceptions of how others would view the subject engaging in specific behaviors [33] such as technology use [22]. These studies have shown that subjective norms are positively related to intention to use a technology, which implies that an SMRA training session that helps patients to think that their family members or health care providers would positively view the patients’ app use is likely to lead the patients to use the app. In sum, TAM could be a useful theoretical framework guiding the design of an SMRA training session that aims to ensure the patients’ app use focusing on the determinants of app use.

In addition to the guiding theoretical framework for the design of the SMRA training session, addressing how to conduct the training session in ways suitable to middle-aged to older patients is important for an appropriate delivery of the training session. Existing studies have indicated that middle-aged to older adults might feel comfortable learning about new technology when (1) training them in small peer groups in a supportive location, (2) providing them with instructions on technology use on a large screen, and (3) providing them with hands-on experience with technology [16,17,34]. In addition, existing studies have indicated that an SMRA training session of approximately 2 hours might be sufficient to help patients become familiar with app use [9,18]. Following these principles, such as conducting an approximately 2-hour small-group SMRA training session at a hospital, the location that advocates patients’ need for health care education [35], and where patients visit for chronic condition management, the training session could be delivered to patients in ways that would help them feel comfortable learning about the app.

**Figure 2:**
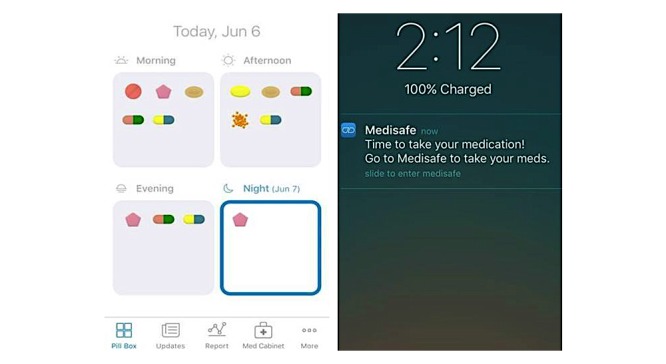
Screenshots of the Medisafe app: virtual pill box (left) and reminder (right) features.

The study reported here, guided by TAM, aimed to (1) assess the feasibility of an SMRA training session designed to target patients’ perceived usefulness of app, perceived ease of app use, and positive subjective norm regarding app use that might lead to their intention to use the app (Figure 1) and (2) gain insight into how to refine the training session in preparation for a larger main study focusing on these theoretical determinants, as well as the practical implications of designing and implementing the training session in a hospital setting.

## Methods

### Pilot Study Design

To meet the first aim of this pilot study, the researchers decided to employ a two-group design with a survey method to (1) have one group receive an SMRA training session designed to target TAM variables (intervention training session) and have another group receive the training session without targeting TAM variables (control training session) and (2) assess the differences in outcome measures (eg, perceived usefulness of SMRA) between the groups [36] so that precisely quantifying whether or not the intervention training session is feasible in targeting TAM variables could be possible [37]. To meet the second aim of the study, the researchers decided to conduct a focus group, which is an appropriate method for exploring shared experiences among a similar group of people [38], to assess why the intervention training session is (or is not) feasible in targeting TAM variables (eg, whether the training session content adequately helped participants perceive the usefulness of SMRA) by exploring participants’ communal perceptions of the SMRA in relation to TAM variables at the end of each training session.

Therefore, in this study, one group, as training group, received an intervention training session that was concluded with a one-time survey and a one-time focus group on one of two different study dates. Another group, as nontraining group, received a control training session that was concluded with a one-time survey and a one-time focus group on another date. The details of intervention and control training sessions are described in the following sections.

### Development of an SMRA Training Session

The researchers selected the Medisafe app (Figure 2) developed by Medisafe Inc for SMRA training sessions. This app was selected because it is available as a free app for both iPhone operating system (iOS, Apple Inc) and Android devices, making it cost-effective and accessible to most smartphone users. Additionally, this app has existing evidence of success with middle-aged to older patients with chronic conditions who used the Medisafe app for 6 months, reporting higher levels of medication adherence compared with those without app use [39].

The training sessions for both the training and nontraining groups were developed following the principles deemed suitable for training middle-aged to older patients to use new technologies in general [16,17,34] and SMRA in particular [9,18]. Specifically, the researchers decided to (1) train participants in small groups at their local hospital, (2) use Microsoft PowerPoint slides to provide them with instructions on Medisafe app use, (3) provide them with hands-on experience with the app, and (4) schedule each training session to be approximately 2 hours.

**Figure 3:**
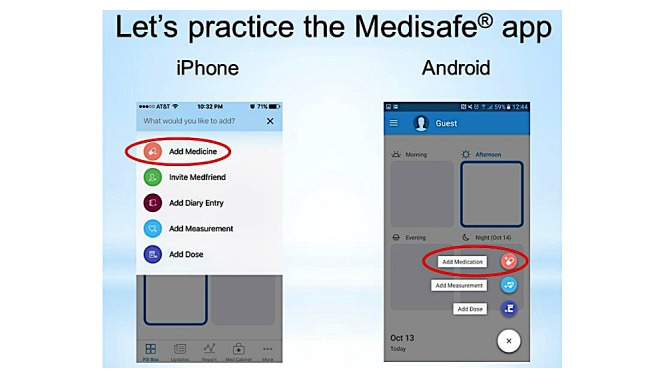
Screenshot of the intervention training session PowerPoint slide.

Regarding intervention training session content for the training group, based on middle-aged to older patients’ perceptions and experiences of SMRA use that previous studies have reported [9,18,19], the researchers developed the content to increase participants’ levels of perceived usefulness of Medisafe app, perceived ease of app use, and positive subjective norm regarding app use. In addition, as iPhone and Android phones differ in the layout of Medisafe app, the researchers developed the content for iPhone users and for Android phone users separately to prevent participants from being confused by instructions on app use that do not correspond with their smartphone version of app (eg, different location of app feature buttons; Figure 3). Regarding control training session content for the nontraining group, the researchers developed content designed to lead participants to explore Medisafe app features on their own (Table 1).

### Sample and Procedures

In fall 2016, this pilot study was conducted at a rural midwestern community hospital after approval was obtained from the university’s institutional review board (IRB) and the hospital’s IRB. To recruit participants, a designated hospital staff member distributed recruitment materials (ie, study description and contact information to pass on to interested patients) via email to health care providers and staff members throughout the hospital.

**Table 1:** Descriptions of intervention training session and control training session.

Training session schedule			Training session content and activity
**Intervention training session for the training group (up to 2 hours)**			
	Introduction of SMRA^a^ (10 min)		Content: introduction of what an SMRA is in general and what the Medisafe app is in particular (eg, who the developer is, where and at what cost and in which languages the app is available to use)
	**Targeting TAM**^b^ **variables (20 min)**		
		Perceived usefulness	Rationale for content: patients reported satisfaction with SMRA that visually (eg, medication pictures) supports correct medication taking [9]; patients described SMRA as useful as it reminds of and helps set up medication routines [19]
			Content: (to increase participants’ levels of perceived usefulness of SMRA) introduction of virtual pill box (ie, feature for visually keeping track of medication list) and reminder (ie, feature for being reminded of taking and refilling medications in a timely fashion) features of the Medisafe app
		Positive subjective norm	Rationale for content: patients’ perceptions of others who support patients’ medication adherence matter to patients’ continuous SMRA use [19]
			Content: (to increase participants’ levels of positive subjective norm regarding SMRA use) introduction of the Medfriend feature (ie, feature for notifying co-app users such as family members if a patient missed a reminder from the app so that they could call or text the patient to additionally remind of medication taking) of the Medisafe app
		Perceived ease of use (hands-on experience with SMRA)	Rationale for content: patients often struggled with navigating SMRA features by missing or misinterpreting app feature buttons [18]
			Content: provision of step-by-step instructions on how to use virtual pill box (eg, adding either prescribed or hypothetical medications to the app) and reminder (eg, scheduling reminders and receiving them from the app) features of the Medisafe app
			Activity: (to increase participants’ levels of perceived ease of SMRA use) participants practice the above features following step-by-step instructions; participants repeat the practice on their own to ensure their competency in app use
	Survey (10 min)		Activity: participants complete a survey measuring TAM variables
	Focus group (1 hour)		Activity: participants describe their perceptions of the Medisafe app in relation to TAM variables
**Control training session for the nontraining group (up to 2 hours)**			
	Introduction of SMRA (10 min)		Content: introduction of what an SMRA is in general and what the Medisafe app is in particular
	Hands-on experience with SMRA (20 min)		Activity: participants explore any Medisafe app features on their own
	Survey (10 min)		Activity: participants complete a survey measuring TAM variables
	Focus group (1 hour)		Activity: participants describe their perceptions of the Medisafe app in relation to TAM variables

^a^SMRA: smartphone medication reminder app.

^b^TAM: technology acceptance model.

Eligible participants for the study were patients who had been managing a chronic condition for at least 3 months preceding the study, were taking at least 3 prescribed medications, were aged 40 years or older, use a smartphone, and had no experience of SMRA use. Hospitalized patients, patients with limited English proficiency, and patients unable to travel to the study location during the study period were excluded.

Interested patients contacted 2 researchers (DP and KH) to participate in the study, and 11 participants were recruited as the final sample. The researchers (DP and KH) asked participants to take part in the study on either of two different study dates at their convenience; they were invited to a private room at the hospital on the date they had chosen. One date was for the intervention training session (for training group) and another date was for the control training session (for nontraining group); participants were not informed which date was for the intervention or control training session.

Participants arrived for their group training session, and after obtaining informed consent, the researchers asked participants to download the Medisafe app to their smartphones. After the participants successfully downloaded the Medisafe app to their smartphones, they either participated in the intervention training session (led by researchers DP and EG on one study date) or the control training session (led by researchers DP and KH on another study date). There was no incentive for the completion of training sessions.

### Data Collection

#### Survey

Following the first part of training sessions (hands-on experience with SMRA), the participants completed a survey questionnaire related to demographics and TAM variables (Multimedia Appendix 1). The following TAM variables were measured on a 7-point Likert-type scale ranging from “strongly disagree” (score 1) to “strongly agree” (score 7), and item scores for each variable were summated and averaged to create variable scales (eg, perceived usefulness scale) for data analysis: perceived usefulness (6 items adapted from Davis’s [20] study; mean=6.73, median=6.83, SD=0.35), perceived ease of use (6 items adapted from Davis’s [20] study; mean=6.05, median=6.33, SD=1.51), subjective norm (7 items adapted from Charng et al’s [40] study; mean=4.55, median=4.43, SD=1.17), and intention to use an SMRA (3 items adapted from Venkatesh et al’s [23] study; mean=6.03, median=6.00, SD=0.81). Cronbach alpha was calculated to assess an internal consistency of each variable scale, and scores on perceived usefulness (Cronbach alpha=.79), perceived ease of use (Cronbach alpha=.99), subjective norm (Cronbach alpha=.88), and intention to use an SMRA (Cronbach alpha=.97) were deemed acceptable.

#### Focus Groups

A focus group followed the survey. Participants were asked to describe their perceptions of the SMRA in relation to TAM variables in depth; a semistructured interview guide focused on (1) participants’ general struggles related to medication adherence, (2) past strategies to address these struggles, and (3) perceptions about using the SMRA both during the study, as well as how they might use it in their real lives. The focus groups were audio-recorded and then transcribed using a transcription service; after transcription was completed, 2 researchers (DP and EG) checked the accuracy of transcripts. The researchers removed any identifying information from the transcripts and replaced participants’ names with pseudonyms.

### Data Analysis

Quantitative data was analyzed using SPSS 16.0 (SPSS Inc). As the assumption for normal distribution of the data was unmet (eg, histogram), a Mann-Whitney *U* test was conducted to assess whether the training group and nontraining group differ in TAM variables.

The transcripts were analyzed using a first- and second-level coding method [38] to identify the themes reflecting participants’ perceptions of the SMRA in relation to TAM variables. Two researchers coded the transcripts independently and compared and combined codes. Following the consolidated criteria for reporting qualitative research guidelines [41], the other two researchers reviewed the codes to minimize bias in coding. In addition, the researchers discussed whether and concluded that saturation had been reached.

## Results

### Participant Characteristics

Of the 11 participants, 45% (5/11) were part of the training group and 55% (6/11) were a part of the nontraining group. All participants were white, and the majority of them were female (73%, 8/11). Participants’ ages ranged from 45 to 70 years (median=58, SD=9.55). The majority of participants reported education levels of bachelor’s degree or higher (64%, 7/11) and annual household income levels of US $90,000 or greater (73%, 8/11). All but one participant reported they had never used an SMRA before. Chi-square tests and Mann-Whitney *U* tests revealed that there were no significant differences in demographics between training group and nontraining group (Table 2).

### Differences in TAM Variables Between Training Group and Nontraining Group

Mann-Whitney *U* tests revealed differences in TAM variables between the training group and nontraining group (Table 3). Although there was no significant difference between the groups (*P*=.33), the training group (median=6.67) reported higher levels of intention to use an SMRA than the nontraining group (median=5.83). In addition, although there was no significant difference between the groups (*P*=.13), the training group (median=7.00) reported higher levels of perceived ease of use than the nontraining group (median=6.25).

The training group (median=6.50) reported lower levels of perceived usefulness than the nontraining group (median=6.92) at the marginally significant level (*U*=4.50, Z=−1.99, *P*=.05). In addition, although there was no significant difference between the groups (*P*=.25), the training group (median=4.29) reported lower levels of positive subjective norm than the nontraining group (median=4.50).

**Table 2:** Demographic profile of the participants.

Demographics		Training group (n=5)	Nontraining group (n=6)	*P* value^a,b^
Age in years, median (SD)		58 (9.33)	60 (10.57)	>.99^a^
**Gender, n (%)**				.06^b^
	Female	5 (100)	3 (50)	
	Male	0 (0)	3 (50)	
**Ethnicity, n (%)**				.34^b^
	Non-Hispanic or Latino	5 (100)	5 (83)	
	Unknown	0 (0)	1 (17)	
**Race, n (%)**				–
	White	5 (100)	6 (100)	
**Education, n (%)**				>.99^a^
	Some college, no degree	1 (20)	0 (0)	
	Associate degree (eg, occupational)	0 (0)	1 (17)	
	Associate degree (academic)	1 (20)	0 (0)	
	Bachelor’s degree	1 (20)	3 (50)	
	Master’s degree	1 (20)	1 (17)	
	Professional school degree	1 (20)	0 (0)	
**Income (USD), n (%)**				.76^a^
	$70,000-$79,999	0 (0)	1 (17)	
	$80,000-$89,999	0 (0)	1 (17)	
	$90,000-$99,999	2 (40)	1 (17)	
	Greater than $100,000	2 (40)	3 (50)	
**Experience of SMRA**^c^ **use, n (%)**				.25^b^
	Yes	1^d^ (20)	0 (0)	
	No	4 (80)	6 (100)	
**Chronic condition, n (%)**				
	Acid reflux	0 (0)	1 (17)	.34^b^
	Anxiety	0 (0)	1 (17)	.34^b^
	Arthritis	2 (40)	4 (67)	.38^b^
	Asthma	1 (20)	0 (0)	.25^b^
	Back pain	1 (20)	2 (33)	.62^b^
	Diabetes	1 (20)	2 (33)	.62^b^
	Epilepsy	1 (20)	0 (0)	.29^b^
	Heart disease	1 (20)	1 (17)	.89^b^
	High blood pressure	2 (40)	4 (67)	.38^b^
	High cholesterol	0 (0)	1 (17)	.34^b^
	Ulcer or stomach disease	1 (20)	0 (0)	.25^b^
**Number of chronic condition, n (%)**				.18^a^
	1	0 (0)	1 (17)	
	2	5 (100)	1 (17)	
	3	0 (0)	3 (50)	
	4	0 (0)	1 (17)	
**Number of prescribed medication, n (%)**				.69^a^
	3	2 (40)	2 (33)	
	4	1 (20)	0 (0)	
	5	1 (20)	1 (17)	
	6	1 (20)	0 (0)	
	8	0 (0)	1 (17)	
**Presence of a caregiver, n (%)**				.75^b^
	Yes	1 (20)	1 (17)	
	No	3 (60)	5 (83)	

^a^*P* values calculated using Mann-Whitney *U* tests.

^b^*P* values calculated using chi-square tests.

^c^SMRA: smartphone medication reminder app.

^d^All participants reported having no experience of SMRA use during the participant recruitment but one of them reported she previously tried and stopped using another SMRA (different from the Medisafe app) during the focus group.

**Table 3:** Differences in technology acceptance model (TAM) variables between training group and nontraining group.

Variables	Interquartile range	Training group (n=5)		Nontraining group (n=6)		*P* value
		Median	Mean (SD)	Median	Mean (SD)	
Intention to use an SMRA^a^	5.00-7.00	6.67	6.33 (0.85)	5.83	5.78 (0.75)	.33
Perceived usefulness	6.50-7.00	6.50	6.50 (0.42)	6.92	6.92 (0.09)	.05
Perceived ease of use	6.00-7.00	7.00	6.73 (0.43)	6.25	5.47 (1.88)	.13
Positive subjective norm	4.14-5.29	4.29	4.09 (1.34)	4.50	4.93 (0.96)	.25

^a^SMRA: smartphone medication reminder app.

### Participants’ Perceptions of the SMRA in Relation to TAM Variables

Throughout the focus groups, participants described their perceptions of the SMRA in relation to TAM variables including perceived usefulness, perceived ease of use, positive subjective norm, and intention to use the app.

#### Perceived Usefulness

Participants described the real-world utility of the SMRA in addressing specific struggles in medication adherence in their lives. Furthermore, participants’ intention to use an SMRA was based upon the degree to which their perceived usefulness was positively rated.

##### Real-World Utility of SMRA in Medication Adherence

Participants described the utility of the SMRA in medication adherence in terms of struggles encountered in the real-world setting. For example, one of participants described her struggle in remembering to take her medications in the evening, saying:

My bedtime one I forget a lot, just because it’s, you know, it’s later in the evening or whatever and I get busy and I forget that one.

Regarding this struggle, she described the fact that SMRA users would receive reminders from the app as helpful for remembering medication taking, noting:

You always have your phone with you...so, the fact that, you know, it would...vibrate [to remind of medication taking] or do whatever you set it to, I mean, I think that would be very helpful.

Another of the participants described struggles in remembering to take medications when traveling, particularly to places with a different time zone. As Susanna said:

If you’re travelling, you know, and you’re in [name of place] where the time changes so drastically...Should I take it at, what I would have taken in [name of place] or do I switch it?

Regarding this struggle, she noted that an SMRA feature that reminds of medication taking based on local time would be helpful as she continued:

Then the app would go off [to remind of medication taking].

Yet another participant described her struggle to remember to refill her prescriptions in a timely fashion:

I thought I had some left but I didn’t have any left. So then it’s like, ok, you gotta call your friends over at the pharmacy, and say, help!

This participant found the Medisafe app feature that reminds users of their medication refilling schedule to be particularly helpful:

The fact that it’s gonna remind me is a, is a big help to me, especially when you get 3 months out it’s, you know, your mind kind of goes, so.

Some of participants described struggling to manage changing medications. For example, William said:

[I] can identify some of them, but, every few years they change...in the last, 3or 4 months I’ve, noticed that, there have been a time or two I forgot them.

He went on to acknowledge the potential value of the SMRA in helping him overcome this struggle:

If me and this phone can become friends we might be able to set up where it could remind me pretty good.

In addition, Emily described an SMRA feature that enables recording medications in the app in visually precise ways (ie, virtual pill box) as helpful for managing varying medications, as she said:

I did like how that had, um, the pills you could put the color and the shape...I thought that was nice. Especially because they do change so often.

A final medication adherence struggle participants identified was managing temporary medications. Although participants described using specific tools (eg, Pill box and Outlook calendar) or setting up rules or habits (eg, brushing teeth after medication taking) as ways to support medication adherence, they agreed that managing temporary medications could be particularly challenging. For example, Avery noted:

One of those things that I don’t normally take, I’ll write right on the bottle...all the dates [for medication taking]...it works but if you have a lot of them it doesn’t.

She perceived the potential value of the app as a convenient way to manage temporary medications, noting:

It’d be a lot more convenient to have it set up in there as a temporary, you know, a temporary dosage.

##### Intention to Use an SMRA

Some of participants described their intention to use an SMRA in relation to perceived usefulness, as William said, when asked if the participants would be willing to use the app in future:

Oh, I think it would be helpful.

More specifically, Grace described her intention to use the reminder feature because it seems helpful for remembering medication taking on weekends when she often ended up second guessing (eg, “Did I take those before we left the house, I don’t remember”). She explained:

Because weekends are hard for me and busy with kids and doing things and so...I think I’ll get in the routine of clicking those reminders...it will be a big help.

In sum, the focus groups revealed the utility of SMRA in real-world medication adherence and indicated a potential positive link between perceived usefulness and intention to use the app.

#### Perceived Ease of Use

The nontraining group described more struggles in SMRA use than the training group. Furthermore, participants’ intention to use an SMRA was based upon the degree to which their perceived ease of use was positively rated.

##### Challenge in SMRA Use by Nontraining Group

The nontraining group, which was given 20 min to explore Medisafe features on their own, described difficulty in using SMRA features that the training group was able to use after 20 min of training that involved step-by-step instruction on app features. Nontraining group participant Emma said:

You can’t sit here in 10,15 minutes and understand what’s going on with that app.

Michael agreed:

I think I’d need more than 20 minutes to really get a feel for it.

Olivia observed:

I had a little trouble navigating...one of the pills [I recorded in the app] was the wrong shape so I had to go in and change...and it didn’t change for me right away.

William said:

I tried to program it [reminder schedule] to 3 a day, but, uh, the milligrams of it [regarding dosing schedule] or whatever, um, never would let me put those in.

In addition, the nontraining group was unaware of SMRA features that were introduced to the training group. Emily said:

I clicked on that [Medfriend feature] but didn’t-didn’t know what it was so I just got out of it.

Michael observed:

I didn’t pay attention to that [virtual pill box].

Nontraining group members also had several unanswered questions for the researchers. Emma, for instance, wanted to know:

What kind of sound does it make when you miss your pill?

Andrew asked:

Can you set the reminders for different days of the week? The times?

##### Intention to Use an SMRA

Some of participants described that their intention to use an SMRA depends on perceived ease of use, as Avery said:

Well, [if] it’s not easy then I’m not gonna deal with it. Forget it.

In addition, Hannah described that she previously tried another SMRA (different from the Medisafe app) and stopped using it because “it wasn’t as user friendly.”

In sum, focus groups indicated the challenges in SMRA use by the nontraining group and a potential positive link between perceived ease of use and intention to use the app.

#### Positive Subjective Norm

Participants described their perceptions of others who might view positively the participants’ SMRA use in the real-world setting, such as family members or health care providers. Furthermore, participants’ intention to use an SMRA was based upon the degree to which their subjective norm was positively rated.

##### Family Members and Health Care Providers

Participants described several situations in which they thought others would positively view participants’ SMRA use. Some of participants described that doctors could find patients’ SMRA use to be positive. For example, Emily explained:

When you go to a doctor, they want to know, you know, what all medicines are you on. I can never remember...that [SMRA feature helping find medication names] will be a very, very nice feature to have.

Furthermore, some participants described that family members could find patients’ SMRA use to be positive. Emily said:

For loved ones too, if anything ever happens to me, they could take my phone...if I end up in the emergency room...they’ll know everything that I take, will be right there on my phone.

In addition, Andrew described that his daughters could find using the Medfriend feature with him to be positive, admitting:

I think my 2 daughters would appreciate knowing that I’m taking my medication.

##### Intention to Use an SMRA

Some of participants described their intention to use an SMRA in relation to subjective norm. For example, Grace described her intention to use an SMRA feature that enables recording a medication list in the app because she thought her doctor would perceive it to be helpful. She said:

I would use it...if I go to an appointment or something, you know, what are you on? I-I can never remember the dosages and things like that so to have it on my phone, it’ll be nice.

In contrast, Chloe described her intention to not use the Medfriend feature because others might feel bothered by using the feature with her, as she said:

No, I don’t think I would need anybody to call me because...they would get, uh, tired of saying, hey, take your medicine. I’m gonna take my medicine, I have to take my medicine.

In sum, focus groups indicated specific health care settings (eg, primary care and routine medical checkups) in which the participants think their family members or health care providers would find value in the participants’ SMRA use. Furthermore, focus groups indicated a potential positive link between positive subjective norm and intention to use an SMRA.

## Discussion

### Feasibility of the Intervention Training Session

One of the primary aims of this pilot study was to assess the feasibility of an SMRA training session designed to increase patients’ intention to use the app through targeting TAM variables. The findings from this pilot study indicated that the intervention training session was feasible in increasing an intention to use an SMRA through targeting perceived ease of use.

Results revealed that the level of perceived ease of use and the level of intention to use an SMRA were higher in the training group than in the nontraining group, and the focus groups indicated that perceived ease of use might lead to intention to use the app. These findings are consistent with existing studies that have ascertained the path from technology training to perceived ease of use to intention to use the technology [28,42]. In addition, these findings expand existing studies on middle-aged to older patients’ SMRA use [9,18,19] by indicating not only the utility of a training session in promoting patients’ app use but the type and focus of a training session that could be a promising intervention to promote patients’ app use: a scheduled small-group training session in a hospital setting focused on helping patients feel at ease navigating and using the app by providing them with step-by-step instructions on and hands-on experience with app features.

### Limitations and Plans for the Future Study

The second aim of this pilot study was to understand how to better design and implement the training session in a hospital setting for the larger main study. There are a couple of limitations of this pilot study that will be addressed in designing the main study.

As the first limitation of this pilot study, the findings indicated that the intervention training session was not feasible in increasing intention to use an SMRA through targeting perceived usefulness and positive subjective norm. Participants indicated in the focus groups that, not surprisingly, the perceived usefulness and positive subjective norm might lead to intention to use an SMRA, consistent with findings from existing TAM literature [22,25,26,28,31,32,43]. However, quantitative results revealed that the level of perceived usefulness and the level of positive subjective norm in the training group did not surpass those in the nontraining group. Understanding the reasons for these findings is important in moving forward to the main study.

First, it may be that the intervention training session did not adequately address perceived usefulness and positive subjective norm, suggesting a need to refine the content of the training session. Beyond the nonsignificant quantitative results related to perceived usefulness and positive subjective norm, the focus group findings may provide some insight. The focus groups revealed that the intervention training session did not include content that would help target perceived usefulness and positive subjective norm.

Regarding perceived usefulness, whereas the intervention training session focused on introducing participants to the technical utility of SMRA in medication adherence (eg, the virtual pill box as a visual aid for correct medication management), throughout the focus groups, the participants described the real-world or practical utility of an SMRA in relation to perceived usefulness. Regarding positive subjective norm, whereas the intervention training session focused on introducing participants to the technical utility of SMRA that enables their family members or health care providers to monitor and support participants’ medication adherence (ie, Medfriend feature), many participants described the real-world reasons behind why their family members or health care providers might have positive views on participants’ app use in relation to positive subjective norm. In other words, participants indicated that family members or health care providers might view participants’ SMRA use more positively because they perceive it to be useful in helping participants stay healthy, both in a day-to-day sense, as well as during an emergency. In sum, it was indicated that introducing participants to the utility of SMRA in medication adherence without applying it to the real-world setting might lead the intervention training session to not adequately target perceived usefulness and positive subjective norm. In this regard, the researchers will plan to refine the content of the intervention training session in ways that (1) emphasize the real-world application of SMRA to addressing patients’ specific struggles in medication adherence in their lives (to better target perceived usefulness) and (2) help the patients to see how their family members or health care providers might benefit from the patients’ app use (to better target positive subjective norm).

In addition, the intervention training session time allocated to targeting TAM variables (approximately 20 min) might be insufficient to target perceived usefulness and positive subjective norm. For example, within 20 min, the training group participants might feel pressed for time for digesting instructions on SMRA features and might pay more attention to how to use app features than whether and how app features could support them and their family members in (participant) medication adherence in the real-world setting. For this pilot study, the researchers allocated more time to quantitative and qualitative assessments (more than an hour) than targeting TAM variables, given that the primary aims of this study were assessing the feasibility of the intervention training session. For future work, the researchers will plan to increase the time for targeting TAM variables to up to 2 hours, following what the existing studies [9,18] have done (eg, allocating up to 2 hours to the completion of app-related tasks) [18], so that the abovementioned potential effects of the training session time on the training session outcomes could be mitigated.

The second limitation of this pilot study is associated with sampling issues. Specifically, small sample size (n=11) might result in underpowered and nonsignificant findings (ie, type II errors) [44] and might be insufficient to ensure internal consistencies of variable scales [45]. In addition, a power analysis for the main study was not feasible because of the sample size [45]. Following Hertzog’s [45] suggestions for a pilot study sample size for the power analysis, the researchers first planned to recruit at least 20 participants in an attempt to have at least 10 in the training group and 10 in the nontraining group. However, meeting this aim was challenging, and it is attributable to a couple of issues.

Recruitment in a hospital setting may have been more successful except for the time constraints of the hospital staff member who assisted with recruiting (ie, the 2 weeks leading up to the study dates she was unavailable). Addressing time constraint issues with health care professionals is imperative to meet the aims of health care education [35], and the researchers will plan to address this in the future by cooperating with multiple health care providers and staff members, who differ in the time availability of recruitment support, to better meet the aim of participant recruitment for the future study. For health care providers with limited time availability of direct recruitment support, following Lorig’s [46] suggestions, the researchers will plan to ask them to (1) permit researchers to place a recruitment poster and sign-up sheet in their waiting room and (2) refer their patients interested in the study to researchers for more information about an SMRA training session.

Furthermore, following what existing studies have done, utilizing more varied recruitment strategies such as (1) placing recruitment flyers in community centers [17,18] or on social media (given the increasing use of social media within the middle-aged to older population) [47], (2) recruiting potential participants at hospital events [24], (3) using the snowball sampling method [48], and (4) using participant incentives (eg, a gift card) [16,18,19] might facilitate meeting the aim of participant recruitment for this pilot study that the researchers will plan to do for the future study.

Utilizing the above recruitment strategies, in addition to increasing participant number, the researchers will plan to increase participant diversity for the future study, given that all participants in this pilot study were white and the majority of them were female and with higher education and income levels. Existing studies have indicated the impact that demographic variables have on patients’ medication adherence behaviors [8,49] and might have on their SMRA use. In this regard, recruiting a larger and more diverse sample, the researchers will plan to assess the feasibility of an SMRA training session in targeting TAM variables for patients across demographics.

### Conclusions

The findings from this pilot study confirm that an SMRA training session for middle-aged to older patients with chronic conditions is important in promoting their app use. Specifically, the value in designing a TAM-based training session was indicated, such that the intervention training session appeared feasible in leading patients to adopt the use of an SMRA by first targeting perceived ease of use, guided by TAM. The findings also provide practical implications that will inform the design of the larger main study. Refining intervention training session content (ie, focusing on the utility of an SMRA in the real-world setting) informed by this study and providing patients with sufficient time for digesting instructions on SMRA features, the training session might better help increase patients’ levels of perceived usefulness and positive subjective norm that might also lead them to adopt the use of an SMRA. In addition, cooperating with multiple health care professionals in participant recruitment could help secure a sufficient and continuous recruitment support for meeting the aim of participant recruitment in a hospital setting.

## Appendix

Multimedia Appendix 1 Measurement items for perceived usefulness, perceived ease of use, subjective norm, and intention to use an SMRA.

## Abbreviations

IRB: institutional review board
SMRA: smartphone medication reminder app
TAM: technology acceptance model
UTAUT: unified theory of acceptance and use of technology
