# Supplementary material for: Implications for Training on Smartphone Medication Reminder App Use by Adults With Chronic Conditions: Pilot Study Applying the Technology Acceptance Model
Source: JMIR Form Res. 2017 Nov 10;1(1):e5. doi: 10.2196/formative.8027 (PMC6334682; doi:10.2196/formative.8027)
Supplement: Multimedia Appendix 1 [file formative_v1i1e5_app1.pdf]

Measurement items for perceived usefulness, perceived ease of use, subjective norm, and intention to use an SMRA[a]

Perceived usefulness: 6 items adapted from Davis's [20] study

1. Using the Medisafe app would help me quickly check what medications I should take.
2. Using the Medisafe app would help me to better manage and keep track of my medications.
3. Using the Medisafe app would help me to not miss taking my medications.
4. Using the Medisafe app would help me to remember to take my medications.
5. Using the Medisafe app would make it easier to manage and keep track of my medications.
6. I would find the Medisafe app to be useful in managing and keeping track of my medications.

Perceived ease of use: 6 items adapted from Davis's [20] study

1. Learning to use the Medisafe app was easy for me.
2. I found it easy to get the Medisafe app to do what I want it to do to manage my medications.
3. Using the Medisafe app was clear and understandable.
4. I found the Medisafe app to be flexible to use.
5. It was easy for me to become skillful at using the Medisafe app.
6. I found the Medisafe app to be easy to use.

Subjective norm: 7 items adapted from Charng et al's [40] study

1. They[b] would think that using the Medisafe app is important to me.
2. It is important to them that I continuously use the Medisafe app.
3. It really would not matter to them if I decided to give up using the Medisafe app (reverse-coded).
4. They would expect me to continuously use the Medisafe app.
5. None of them would really be surprised if I just stopped using the Medisafe app (reverse-coded).
6. They would probably be disappointed in me if I just decided to stop using the Medisafe app.
7. They would probably make me feel guilty if I quit using the Medisafe app.

Intention to use an SMRA: 3 items adapted from Venkatesh et al's [23] study

1. I intend to use the Medisafe app in the next months.
2. I predict I would use the Medisafe app in the next months.
3. I plan to use the Medisafe app in the next months.

[a]All items were measured on a 7-point Likert-type scale ranging from "strongly disagree" (score 1) to "strongly agree" (score 7).
[b]"They" referred to "family members and healthcare providers" in the survey questionnaire.
